# Acylated ghrelin and leptin concentrations in patients with type 2 diabetes mellitus, people with prediabetes and first degree relatives of patients with diabetes, a comparative study

**DOI:** 10.1186/2251-6581-12-51

**Published:** 2013-12-19

**Authors:** Faranak Sharifi, Mahdi Yamini, Abdolreza Esmaeilzadeh, Nouraddin Mousavinasab, Zahra Shajari

**Affiliations:** 1 Professor in Clinical Endocrinology, Metabolic Diseases Research Center, Zanjan University of Medical Sciences, Zanjan, Iran; 2 Resident in Internal Medicine, Zanjan University of Medical Sciences, Zanjan, Iran; 3 Clinical Immunology, Zanjan University of Medical Sciences, Zanjan, Iran; 4 Medical Statistics, Metabolic Disease Research Center, Zanjan University of Medical Sciences, Zanjan, Iran; 5 Scientific Researcher, Metabolic Disease Research Center, Zanjan University of Medical Sciences, Zanjan, Iran

**Keywords:** Acylated ghrelin, Diabetes mellitus, Leptin, Prediabete

## Abstract

**Background:**

Ghrelin is known as a new endocrine component supposed to have an influence in control of feeding behavior and energy balance. Recent studies have shown that ghrelin concentration in the subjects with diabetes mellitus type 2 (DM 2) is lower than normal. To clarify the relationship between ghrelin and insulin resistance and also DM 2, a cross-sectional study was designed.

**Methods:**

In a cross-sectional study, 87 subjects were enrolled in three groups, 29 with DM2, 29 pre-diabetes state and 29 normoglycemic subjects of first-degree relatives of diabetic group. After clinical examination, blood samples were taken to measure fasting blood glucose, HbA1c, lipids, insulin, leptin and acylated ghrelin concentrations.

**Results:**

Mean serum concentrations of acylated ghrelin in all groups (47.4 ± 27.9 pg/ml) were lower than normal values (*150.3 ± 56.4 pg/ml*) (P: 0.006) without significant difference within groups comparison(P: 0.1). A significant correlation was found between ghrelin concentration with body mass index (BMI) (r: -0.23, p <0.02) and abdominal circumference (AC) (r: -0.28, P < 0.008). Also inverse relationship between ghrelin level and insulin resistance (HOMA-IR) (r: -.032, p: 0.002) was seen in all subjects. Leptin level has a significant correlation with abdominal circumference (AC) and BMI (P < 0.0001) but not with ghrelin.

**Conclusion:**

This study showed that obesity has a strong association with the reduced level of ghrelin concentration. It seems that the process of ghrelin reduction is initiated in earlier stages of insulin resistance prior to the onset of overt DM.

## Background

Diabetes mellitus (DM) is a heterogeneous disease that is characterized by hyperglycemia and glucose intolerance [1, 2]. Given the high prevalence of DM, identifying the factors that may affect its process and cause the pathological changes in the body is important [3]. Nowadays a lot of changes in different hormones and cytokines have been reported in DM that one of them is in a hormone called ghrelin [3].

Ghrelin is known as a new endocrine pathway in the control of feeding behavior and energy balance in the last decade. Ghrelin is a 28 amino acid hormone is secreted by many tissues, but its main source is the gastric mucosa [4–7]. Active form of ghrelin is acylated ghrelin with some metabolic actions like stimulation the appetite, increase the secretion of growth hormone, decrease insulin secretion from the pancreas, reducing in energy consumption by the body and effects on growth and peripheral metabolism especially of fats and carbohydrates [8–11]. Ghrelin effects are mediated by growth hormone receptor (GHS-R) in pancreatic islets cells, hypothalamus - pituitary axis, kidney, small intestine and placenta and stimulates growth hormone secretion and inhibits the release of calcium and insulin from pancreas.

Reduction of electrical activity in β-cells is another possible mechanism of action for ghrelin. Low ghrelin levels are associated with high levels of insulin and insulin resistance [12]. On the other hand the plasma ghrelin levels rise in malnutrition, cachexia and anorexia nervosa while the levels are reduced in obesity. Lower ghrelin concentration in obesity have attributed to higher insulin concentration, also lower ghrelin levels after eating attributed to increased insulin levels after carbohydrate intake in some studies. It has been reported that injection of insulin in type 1 DM makes some reduction in ghrelin concentration.

Recent studies have shown that ghrelin concentration in subjects with DM2 is lower than normal [3]. Some studies supports the hypothesis that low ghrelin level can be considered as a risk factor of diabetes mellitus developing [12]. It's not clear whether low ghrelin level is a risk factor for DM2 and initiates before developing DM or is secondary to it [13].

Leptin is a hormone that works as a mediator in the stomach – hypothalamus pathway and provides information about the body's energy storage in adipocytes. Also, its level is associated with obesity [3]. Leptin reduces the amount of ghrelin secreted from the stomach. On the other hand in an in vitro study on rat gastric mucosa, leptin injections caused ghrelin suppression in overweight rats without any effect in normal- weight rats [3].

Whether low concentration of ghrelin is a primary or a secondary event in DM2 is unclear and also its relationship with leptin is ambiguous. So this study was designed to describe acylated ghrelin and leptin concentrations in people with different degrees of insulin resistance and dysglycemia including patients with new-onset DM2, people with impaired fasting glucose (IFG)(prediabetes situation) and those normoglycemic first-degree relatives of patients with DM2. We also aimed to determine the relationship between acylated ghrelin, leptin and insulin resistance in the study groups.

## Methods

### Subjects

This study was conducted on eighty-seven people who were sequentially selected from those referred to Vali-asr hospital, an academic hospital; in Zanjan, Iran in 2012.

Twenty-nine newly diagnosed DM2 subjects (one year or less) were put into the first group. Twenty-nine with fasting plasma glucose (FPG) concentration between 100 and 126 mg/dl confirmed twice in repeated measurements or those with impaired glucose tolerance (IGT) defined by ADA criteria (Two-hour plasma glucose 140–200 mg/dL during a75 gr oral anhydrous glucose tolerance test )were considered as prediabetes group 2.

Offspring of patients with DM2 who were more than twenty years old were invited and checked for their FPG and those who were normoglycemic (FPG <100 mg/dl) were entered in third group.

Who had history of using oral hypoglycemic agents or insulin, medications that affect blood lipids or insulin levels, supplements and appetite altering drugs, heart failure, liver or renal failure and acute or chronic inflammatory disorders were excluded from the study.

This study was approved by ethical committee of Zanjan University of Medical Sciences. All the participants were informed about the aims of study and written consent were obtained from all of them.

### Measurements

Height, weight and waist circumference of the patients were measured by standard methods and body mass index (BMI) was calculated for all the participants. Blood pressure was measured two times with a 15- minute interval in sitting position and the mean of them was recorded for each of the patients and pressures equal to or greater than 140/90 mm Hg on two occasions were considered as hypertension. People with body mass index (BMI) higher than 30 kg/m2 considered obese and those with BMI between 25 to 30 kg/m^2^ were considered as overweight. Abdominal obesity was defined as abdominal circumference greater than 102 cm in men and 88 cm in women. Waist to hip ratio (WHR) greater than 1 in men and 0.8 in women was considered high.

Blood samples were taken after at least 12 hours of fasting in all the participants. Plasma glucose was measured by the glucose-peroxidase colorimetric enzymatic method with a sensitivity of 5 mg/dl .Serum Cholesterol and Triglyceride and High density lipoprotein cholesterol (HDL-C) of all the participants were measured enzymatically (Pars azmoon kit, Iran). Also HbA1c was checked for all the participants using Nycocard method.

Fasting serum Insulin levels were measured with electrochemiluminescence immunoassay (ECLIA) using commertial kits (Roche, German), with sensitivity of 0.75 μiu/ml (normal range:0.7-25 μiu/ml). To calculate insulin resistance, HOMA-IR was used based on the formula of glucose × insulin/405 and values higher than 2.1 was considered insulin resistance [14].

To acquire accurate data on ghrelin concentrations, a standard procedure for the collection of blood samples was used including collection of blood samples with EDTA–aprotinin ,keep them chilled and centrifuge as soon as possible, within 30 min after the collection; and acidification with 1 mol/L HCl (10% of sample volume) added to the plasma sample for adjustment to pH 4 to preserve plasma acylated ghrelin.

Plasma acylated-ghrelin and leptin were determined using the Elisa kit from BioVendor Laboratory Medicine Industry (Czech Republic). These tests were done with long immunological reaction method (incubation 4 to 20 hours) to achieve maximum sensitivity of 0.3 pg/ml and 0.2 pg/ml for acylated ghrelin and leptin respectively. Intra assay and inter assay CV of the kit to measure acylated ghrelin was 1.8% and 3.8% respectively. Intra assay and inter assay CV of the leptin kit was 2.4% and 5.4% respectively.

### Statistical analysis

Data were analyzed with SPSS version 16.0 software. Descriptive statistics such as mean, median and standard deviation were used to describe the statistics. ANOVA was used to compare the groups for quantitative and chi-square test was used for qualitative variables. To assess the relationship between the variables simple and multivariate regression analysis were used.

## Results

87 eligible patients were assigned in 3 groups as follows: 29 subjects with diabetes mellitus type 2 in group one, 29 patients with IFG or IGT in group two, and 29 participants with normoglycemic status in group three.

The mean age of the first group was 47.8 ± 11.6 years and in the second group was 53.3 ± 13.2 years. Although no significant difference was found between diabetic and prediabetic patients for their age, normoglycemic subjects in the third group were significantly younger (35.4 ± 2.9, p < 0.0001). There were no significant differences within three groups by sex. Clinical and laboratory characteristics of the participants have been illustrated in Table 1.

**Table 1:** **Comparison of clinical characteristics of people in three study groups**

Clinical variables	Group1: DM		Group 2: pre-DM		Group 3:		p
	N: 29		N: 29		Normoglycemic		
					N: 29		
Age (years)	47.8 ± 11.6		53.5 ± 13.2		35.4 ± 9.2		0.0001^1^
Weight (kg)	78.2 ± 18.2		73.7 ± 13.2		77.3 ± 14.1		0.521
BMI (kg/m2)	29.2 ± 5.4		27.8 ± 4.5		28.8 ± 4.3		0.548
WHR	0.88 ± 0.08		0.86 ± 0.06		0.83 ± 0.05		0.009^1^
AC (cm)	106.8 ± 13.5		102.7 ± 9.9		103.3 ± 11.2		0.365
SBP	129.6 ± 18.6		130 ± 17.3		120.1 ± 13.3		0.04^1^
DBP	78.5 ± 11.5		76.4 ± 9.2		73.2 ± 9.2		0.138
**Laboratory variable**	**Mean ± SD**	**median**	**Mean ± SD**	**median**	**Mean ± SD**	**median**	**P**
acylated ghrelin (pg/ml)	45.3 ± 33.2	40.1	45.5 ± 10.5	44.9	51.5 ± 40.2	56.5	0.1
Leptin (ng/ml)	15.2 ± 14	14.3	13.7 ± 10.5	13.2	16.2 ± 9.5	18.3	0.1
Insulin (μIU/ml)	5.5 ± 7.5	4.2	6.7 ± 5.3	5	6.8 ± 7.8	6.7	0.18
HOMA-IR	2.1 ± 2.5	2.3	1.7 ± 1.4	1.6	1.4 ± 1.6	1.5	0.4
Total Cholesterol (mg/dl)	188.3 ± 31.4	193	190 ± 45.6	196	172.8 ± 20.1	178	0.1
TG (mg/ dl)	190.9 ± 91.7	181	169 ± 82	174	104 ± 44.5	105	<0.0001
HDL (mg/ dl)	46.5 ± 6.4	45	43.3 ± 7.9	48	52.2 ± 3.9	56	<0.0001
LDL (mg/ dl)	103 ± 35.5	104	113.2 ± 38.7	113	99.7 ± 17.2	97	0.2

BMI: Body mass index, WHR: waist to hip ratio, AC: abdomen circumference, SBP: systolic blood pressure, DBP: diastolic blood pressure, TG: triglycerid.

CHOL: total cholestrole, LDL: low density lipoprotein.

Pvalue <0.05 is considered statistically significant.

Table 1 shows that clinical variables including weight, BMI, abdominal circumference (AC) and diastolic blood pressure was not statistically significant, while systolic blood pressure in prediabetic group was significantly higher than in normoglycemic subjects (P: 0.04) (group 3). The prevalence of hypertension (HTN) in the diabetic group (34.5%) was higher than the pre-diabetes group (27.6%) and normal relatives (3.4%) (P: 0.004).

As illustrated in Table 1, triglyceride (TG ) concentration in the diabetic group was higher than two other (p < 0.0001). Although HDL-c concentrations were lower in diabetic and pre-diabetic subjects compared with normal individuals (p < 0.0001), the mean levels of LDL-c had no significant differences among the three groups. The prevalence of hypercholesterolemia in diabetic group was significantly higher than the pre-diabetic and also normoglycemic subjects ( p: 0.008). Mean of serum insulin and HOMA-IR was not statistically different among the three groups (Table 1), but it was higher among obese subjects (9.3 ± 9.1 pg/ml) rather than overweight (5.7 ± 5.1 pg/ml) and normal BMI participants (3.4 ± 2.1 pg/ml) (p: 0.009). Serum leptin was not statistically different within the three groups (Table 1).

Acylated ghrelin concentrations in all three groups (47.4 ± 27.9 pg/ml) were lower than normal values (199 ± 23 *pg/ml*) (P: 0.006). A serum concentration of acylated ghrelin was not statistically different within groups’ comparison (p:0.1). Although no significant difference was found between acylated ghrelin levels with obesity, overweight and normal BMI subjects in total subjects, acylated ghrelin levels was lower in obese subjects of third group (P: 0.04) (Table 2). On the other hand, no significant difference was observed between obese subjects in the three groups for their mean of serum concentrations of acylated ghrelin (P: 0.2). However, A significant inverse relationship was found between serum ghrelin concentration and abdominal circumference (r: - 0.449, P: 0.01), BMI (r:- 0.383, P: 0.04) and waist circumference (r: - 0.464, P: 0.01) in diabetic group. Table 3 shows the relationship between serum acylated ghrelin concentration and other variables like obesity components in the three study groups. The inverse relation of acylated ghrelin and BMI, abdominal or waist circumference remain significant after adjusting for other confounding factors in all the three groups (Table 4).

**Table 2:** **Comparison of the three studied groups for acylated ghrelin levels based on their BMI**

Acylated ghrelin	Group1			Group 2			Group 3		
	(DM)			(pre-DM)			(normoglycemic )		
	N:29			N:29			N:29		
	Normal BMI	Overweight	Obese	Normal BMI	Overweight	Obese	Normal BMI	Overweight	Obese
Mean	72.8	41.6	33.6	46.0	44.6	44.6	53.6	61.5	38.6
(pg/ml)	±62.3	±20.3	±7.3	±15.1	±12.7	±24.2	±6.1	±58.4	±10.3
Median	51.4	28.8	29.7	44.9	40.9	37.8	56.8	48.6	39.7
P	0.07			0.75			0.04		

P value <0.05 is considered statistically significant.

**Table 3:** **The relationship between acylated ghrelin levels with other variables in the three groups**

Groups	Variables		DBP	SBP	insulin	leptin	FBS	Waist	AC	WHR	BMI
Group1	Acylated ghrelin	r	-0.21	-0.140	-0.402	-0.241	0.04	-0.464	-0.449	-0.151	-0.383
(DM)											
N:29		p	0.25	0.43	0.03	0.20	0.81	0.01	0.01	0.43	0.04
Group 2	Acylated ghrelin	r	-0.06	-0.08	-0.205	-0.017	-0.150	-0.554	-0.314	-0.350	-0.181
(pre-DM)											
N:29		p	0.73	0.63	0.28	0.93	0.43	0.002	0.09	0.06	0.34
Group 3	Acylated ghrelin	r	-0.034	-0.311	-0.201	-0.242	-0.099	-0.500	-0.434	-0.151	-0.572
(normoglycemic)											
N:29		p	-0.86	0.10	0.29	-0.20	0.61	0.02	0.01	0.42	0.03

BMI: Body mass index, WHR: waist to hip ratio, AC: abdomen circumference, SBP: systolic blood pressure, DBP: diastolic blood pressure, FBS: Fasting Blood Sugar.

**Table 4:** **Result of multivariate regression analysis acylated ghrelin with waist circumference (waist), abdominal circumference (AC) and BMI and WHR after removing the confounding factor of insulin,age and sex**

		AC	BMI	WHR	Waist
*Insulin is considered as confounding factor*	**r**	-0.23	-0.07	-0.28	-0.28
**Acylated ghrelin**					
	**p**	0.02	0.4	0.008	0.07
*Age and sex are added as confounding factor* s					
**Acylated ghrelin**	**r**	-0.34	-0.31	-0.02	-0.11
	**P**	0.001	0.003	0.8	0.08

BMI: Body mass index, WHR: waist to hip ratio, AC: abdomen circumference.

There was an inverse relationship between acylated ghrelin and insulin levels (r:- 0.402, P: 0.03) In the diabetic group that was attenuated after adjusting for confounding factors like age, sex, waist circumference and BMI (P: 0.5).Also the inverse relationship between ghrelin and leptin concentration which was found in all the study groups was disappeared after adjusting age, sex, waist circumference and BMI.

In all subjects, a direct relationship between leptin levels with BMI and abdominal circumference was observed (P: 0.001). The relationship between ghrelin, leptin and insulin with other variables among three groups are summarized in Table 5.

**Table 5:** **The relationship between ghrelin, leptin and insulin, with all other variables in all subjects**

		DBP	SBP	HDL	LDL	CHOl	TG	HOMA -IR	insulin	ghrelin	leptin	FPG	WC	AC	WHR	BMI
Ghrelin	r	-0.1	-0.16	-0.12	-0.15	-0.08	-0.1	-0.32	-0.23	1	-0.1	-0.14	-0.51	-0.41	-0.2	-0.36
	p	0.32	0.12	0.26	0.15	0.41	0.17	0.002	0.02	_	0.19	0.19	0.001	0.001	0.06	0.001
Leptin	r	0.26	0.22	0.17	0.19	0.24	0.03	0.19	-0.23	-0.1	1	-0.19	0.23	0.52	-0.3	0.6
	p	0.01	0.03	0.1	0.07	0.02	0.72	0.06	0.02	0.19	_	0.07	0.02	0.001	0.3	0.001
Insulin	r	-0.02	0.12	0.1	0.12	0.1	-0.01	0.95	1	-0.2	0.26	-0.92	0.39	0.25	0.15	0.32
	p	0.85	0.26	0.35	0.26	0.33	0.87	0.0001	_	0.02	0.01	0.39	0.001	0.01	0.16	0.002

BMI: Body mass index, TG: triglyceride, WHR: waist to hip ratio CHOL: total cholesterol, AC: abdominal circumference, LDL: low density lipoprotein, SBP: systolic blood pressure, WC: Waist Circumference, HDL: high density lipoprotein.

## Discussion

This study showed that acylated ghrelin levels in all the subjects including patients with overt DM, subjects in prediabetes stage and also first degree relatives of patients with diabetes who are normoglycemic, are lower than the normal values. However, no significant difference was found between the three study groups for their mean serum concentrations of acylated ghrelin. On the other hand, acylated ghrelin concentration was more related to body mass index and abdominal circumference than hyperglycemia itself.

In Study of Monty et al. [15] an inverse relationship between ghrelin levels, BMI and waist circumference was reported that was in agreement of our study results. In our study although a strong inverse correlation between mean concentrations of ghrelin, mean levels of BMI and abdominal circumference was found in all the groups when we ranked subjects based on their BMI, no difference was found between the obese ,overweight and normal weight subjects for the ghrelin levels except for group 3. In group3 obese individuals had lower ghrelin levels than normal weight subjects, this issue also was observed in diabetic patients with P: 0.07, which is close to significance and appears to be significant in higher sample sizes.

Edrmann et al. [3] studied the effect of weight on ghrelin levels and found that ghrelin levels were lower in obese diabetics compared to normal obese individuals. They attributed low levels of ghrelin in obese patients to the effects of insulin, whereas in our study, after adjustment for insulin effects as a confounding factor the inverse relationship between acylated ghrelin with overall and abdominal obesity remained significant.

According to previous studies, ghrelin is one of the factors that are involved in appetite regulation [16, 17] and acts as an appetite stimulating factor to pass starvation messages to brain. So its reduction in obesity can be considered as a defense mechanism of body to decrease appetite. In fact, ghrelin is a natural antagonist of leptin [18]. It seems that reduction in ghrelin in obesity is independent of the influence on insulin.

Seppo et al. [13] in their study found that ghrelin levels are inversely related to the blood glucose level and also reported lower ghrelin levels in patients with type 2 DM. they exposed to discussion this question: Whether low ghrelin levels in type 2 DM is primary or secondary to the disease?

Our study showed that serum ghrelin concentrations even in normoglycemic, insulin resistant people (group 3) are significantly lower than normal ranges reported previously for general population. This issue probably shows that the onset of decline in acylated ghrelin levels may be prior to the onset of hyperglycemia. However, absence of normal control group without insulin resistance in our study may limit this conclusion.

Poyoko et al. [19], in their study found that there are an inverse relationship between Ghrelin and IGF-1 levels with insulin resistance in type 2 diabetes in middle-aged subjects. We also obtained an inverse relationship between acylated ghrelin levels with insulin resistance in all subjects but after removing the effects of obesity, this relationship did not remain significant. This issue may propose a key role of obesity in ghrelin decline and insulin resistance [20]. Furthermore, we found that the inverse relationship between ghrelin and leptin concentrations is due to obesity and after elimination the effect of obesity there was no significant association between the two mediators. This result also confirms the role of obesity as a main factor to change the mediators like ghrelin and leptin.

Seppo et al. [13] proposed low ghrelin level as a risk factor for hypertension, whereas in our study, no significant correlation was found between systolic and diastolic blood pressure and ghrelin concentration out of the effect of age and BMI.

It seems that changes in some mediators secondary to obesity as a main trigger leads to hyperglycemia that can make changes in HDL-C and TG levels in blood.

Our study had some limitations; the first was low sample size in each group that was due to low access to subjects who was eligible for the study. So any interpretation should be made with caution. The second limitation was absence of normal control group without risk factors like relation to diabetic patients, to compare their serum acylated ghrelin concentrations. We relied on normal ranges reported in previous studies for general population, but this may be misleading because of difference in genetic and racial characteristics of the population.

## Conclusions

The results of this study showed that ghrelin concentrations decreases prior to the onset of hyperglycemia and are more related to the fat pad of the body. Longitudinal studies with larger sample size would help to clarify aspects of the subject.
